# Modeling the risk factors for dyslipidemia and blood lipid indices: Ravansar cohort study

**DOI:** 10.1186/s12944-020-01354-z

**Published:** 2020-07-28

**Authors:** Mansour Rezaei, Negin Fakhri, Yahya Pasdar, Mehdi Moradinazar, Farid Najafi

**Affiliations:** 1 Professor of Biostatistics, Biostatistics Department, Social Development and Health Promotion Research Center, Kermanshah University of medical sciences, Kermanshah, Iran; 2 Master of Biostatistics, Student’s research committee, Faculty of Health, Kermanshah University of medical sciences, Kermanshah, Iran; 3 Nutritional Sciences Department, School of Public Health, Kermanshah University of Medical Sciences, Kermanshah, Iran; 4 Social Development and Health Promotion Research Center, Kermanshah University of Medical Sciences, Kermanshah, Iran; 5 Professor of Epidemiology, Research Center for Environmental Determinants of Health, School of Public Health, Kermanshah University of Medical Sciences, Kermanshah, Iran

**Keywords:** Dyslipidemia, Nutrition, Physical status, Blood lipid markers, Artificial neural network, Regression, Cohort study

## Abstract

**Background:**

Lipid disorder is one of the most important risk factors for chronic diseases. Identifying the factors affecting the development of lipid disorders helps reduce chronic diseases, especially Chronic Heart Disease (CHD). The aim of this study was to model the risk factors for dyslipidemia and blood lipid indices.

**Methods:**

This study was conducted based on the data collected in the initial phase of Ravansar cohort study (2014–16). At the beginning, all the 453 available variables were examined in 33 stages of sensitivity analysis by perceptron Artificial Neural Network (ANN) data mining model. In each stage, the variables that were more important in the diagnosis of dyslipidemia were identified. The relationship among the variables was investigated using stepwise regression. The data obtained were analyzed in SPSS software version 25, at 0.05 level of significance.

**Results:**

Forty percent of the subjects were diagnosed with lipid disorder. ANN identified 12 predictor variables for dyslipidemia related to nutrition and physical status. Alkaline phosphatase, Fat Free Mass (FFM) index, and Hemoglobin (HGB) had a significant relationship with all the seven blood lipid markers. The Waist Hip Ratio was the most effective variable that showed a stronger correlation with cholesterol and Low-Density Lipid (LDL). The FFM index had the greatest effect on triglyceride, High-Density Lipid (HDL), cholesterol/HDL, triglyceride/HDL, and LDL/HDL. The greatest coefficients of determination pertained to the triglyceride/HDL (0.203) and cholesterol/HDL (0.188) model with nine variables and the LDL/HDL (0.180) model with eight variables.

**Conclusion:**

According to the results, alkaline phosphatase, FFM index, and HGB were three common predictor variables for all the blood lipid markers. Specialists should focus on controlling these factors in order to gain greater control over blood lipid markers.

## Background

The metabolism of fat in the human body is important because of its association with the health of the circulatory and cardiac system. The significance of dyslipidemia is due to its major role in the development of Coronary Artery Disease (CAD). A total of 38% of deaths are attributed to cardiovascular diseases [1] and the prevalence of dyslipidemia was 34.0% in the study by Pan et al. in 2016 [2]. In Iran, Moradinazar et al. [3] found a prevalence of 40% for dyslipidemia which was similar to the results of other studies reported in the literature (varying between 14 and 79%) [4, 5]. It is also noteworthy that a significant percentage of strokes are due to atherosclerosis. Each dyslipidemia marker has its own impact on the development of chronic diseases, as previous studies have shown that lipid disorder is a known risk factor for Chronic Heart Disease (CHD) [6]. A high level of Low-Density Lipid (LDL) is also an important risk factor for the development and exacerbation of atherosclerosis [7]. Although considered a weak and independent risk factor, hypertriglyceridemia has been raised as a strong independent risk factor for ischemic disease in some recent studies [8]. Moreover, High-Density Lipid (HDL) is proposed as a protective factor against CHD [9, 10]. Considering the dangerous consequences of dyslipidemia and given that it is a controllable risk factor for CHD [11], plus the fact that its prevalence varies according to ethnic, social, economic and cultural characteristics [12–17], the severity and importance of its contributing factors also vary in different societies. Various contributing factors have already been identified for dyslipidemia, but many others remain unknown. Previous studies have shown that lipid disorders may be affected by factors such as nutrition, obesity, physical activity, drug use, and genetics [18–20]. Therefore, by maintaining a balanced diet and physical condition, some lipid disorders can be prevented. Consequently, identifying the contributing factors of dyslipidemia and attempting to control serum cholesterol levels are necessary steps. Data mining methods are a statistical technique used for exploring data. An Artificial Neural Network (ANN) is a data mining method used to identify the most important factors among a set of different factors. The aim of this study was to determine the various factors associated with the incidence of dyslipidemia and cholesterol levels using ANN and multiple regression in the participants of Ravansar cohort.

## Methods

### Study population

The data required for this research has been derived from the recruitment phase of RaNCD cohort study –part of the Prospective Epidemiological Research Studies in IrAN (PERSIAN) – on the residents of Ravansar aged 35 to 65 years. The PERSIAN cohort included 19 cohort studies conducted in different regions of Iran that cover a wide range of Iranians with different ethnicities. The objective of the study was to carry out a 15-year follow up on all the participants. For further details, refer to the protocol and research guide. 15,000 people aged 35–65 years were living in both urban and rural areas of Ravansar district. The 10,000 people based on all available resources and in agreement with the central PERSIAN team included to the study. To increase the feasibility of the study, the samples were recruited from both urban and rural areas. The sample size of the study was proportional to the total population covered by each health center [21].

### Inclusion and exclusion criteria

The inclusion criteria were: Being a resident aged 35–65 years who has lived in the same region for at least one year and has lived in their respective city for at least nine months, willingness to participate and complete the research, signing informed written consent letters, and being capable of communicating with the research team. Since dyslipidemia is a predictor of hypertension and cardiovascular disease [22], the data pertaining to the subjects with cardiovascular disease (10%), hypertension (11%), diabetes (8%), and cancer (1%) was excluded. A total of 7036 subjects were selected of the 10,065 participants of the cohort study.

### Definition and measurements

A BIA device (InBody 770 BIOSPACE, Korea) was used for weight measurement. The height was measured with a precision of 0.1 using a stadiometer. The Body Mass Index (BMI) was calculated by dividing weight (kg) by squared height (m^2^). According to the BMI obtained, the subjects with BMI < 18.5, 18.5 < BMI < 24.9, 25.0 < BMI < 29.9, and BMI ≥30 were categorized as low-weight, normal, overweight, and obese, respectively. The section of the Glasgow Coma Scale (GCS) dealing with activity was used for assessing physical activity. Physical activity levels were classified as low (24–36.5 h per week), moderate (36.6–44.4 h per week) and heavy (≥ 44.5 h per week). Physical activity was also measured based on the 24-h physical activity and a 22-item questionnaire that assessed physical activity as low, moderate, and severe and also considered work and leisure time in one week based on METs/hour/day. The quality of nutrition was assessed based on the Healthy Eating Index - 2015 (HEI-2015), which evaluates 13 food groups; nine for adequate consumption and four for moderate consumption. The HEI score is between 0 and 100, and a high score shows a better quality of nutrition.

Blood parameters, including Total Cholesterol (TC), Triglyceride (TG), HDL, LDL, Gamma glutamyl transferase (GGT), SGPT-ALT, alkaline phosphatase, Hemoglobin (HGB) and creatinine, were measured after eight hours of fasting and the results were categorized according to the WHO guidelines. The levels of carbohydrates, selenium, magnesium (Mg), copper (Cu) and vitamin B12 intake were measured based on the Food Frequency Questionnaire (FFQ). The FFQ was designed to capture eating behaviors in the Italian population [23], and Studies by Troeschel et al. [24] and Newby et al. [25] revealed the good reproducibility and validity of the FFQ. Dyslipidemia was also defined as total cholesterol ≥240 mg/dL [26], LDL cholesterol > 160 mg/dL, or HDL cholesterol < 40 mg/dL or triglyceride > 200 mg/dL [27]. In addition, three cholesterol factors were considered in the evaluations, including TC/HDL, TG/HDL, and LDL/HDL. Smoking habits were assessed as self-reports based on the National Health Insurance Scheme (NHIS). The subjects were divided into three groups, including smokers, non-smokers and former smokers. Smoking habit included the number of cigarettes smoked per day and the duration of smoking in year [3]. Socio-Economic Status (SES) was also determined using the welfare index. The welfare index was calculated by Principal Component Analysis (PCA) of the data related to durable goods, house features, and other facilities.

### Ethical considerations

This study was approved by the Ethics Committee of the Deputy of Research and Technology of Kermanshah University of Medical Sciences (KUMS.REC.1394.315) and a signed consent letter was taken from all the participants.

### Statistical analysis

After manual data modification and re-organization, the perceptron ANN data mining model was used to identify the most important variables affecting dyslipidemia. At the beginning, all the 453 variables, including demographic variables, laboratory parameters, physical activity, nutrition status, lifestyle, etc., were examined using the ANN method in SPSS software version 25. Of all the variables, the most important ones (i.e., the variables that were most crucial for the diagnosis of dyslipidemia) were identified by the 33-step ANN sensitivity analysis. In each step, the sensitivity analysis specified the standardized importance rate for each variable with regard to its effect in predicting dyslipidemia. Then, in accordance with the importance of the variables, 10% of the least important variables were deleted in each step and the sensitivity analysis was performed again on the remaining variables. Finally, 12 significant variables were identified as the predictors of dyslipidemia.

After the review of literature and based on the current researchers’ experience and opinion, two other variables, including the Healthy Eating Index (HEI) and physical activity level, were also added to the analysis. Then, the relationship between these 14 variables and the seven lipid markers (i.e. TC, TG, HDL, LDL, TC/HDL, TG/HDL, and LDL/HDL) was examined using the stepwise multiple regression model. Only 1% of the subjects had missing information and their data were excluded from the ANN analysis but included in the multiple regression analysis. The data obtained were analyzed in SPSS software version 25, at a significance level of 0.05.

#### Results

Of the total of 10,065 samples in the cohort study, 7036 subject (51.1% male and 48.9% female) aged 35 to 65 years were examined in this research, among whom 40.4% (*n* = 2844) had lipid disorder, including 50.1% of the men and 30.3% of the women. Sex, marital status, education level, BMI and smoking status had significant relationships with dyslipidemia (Table 1).

**Table 1:** A comparison of the frequency of the demographic characteristics (%) by dyslipidemia status

Variable		Dyslipidemia		Total N (%)	P^*^
		Yes	No		
**Sex**	**Female**	1043 (30.3)	2399 (69.7)	3442 (48.9)	<0.001
	**Male**	1801 (50.1)	1791 (49.9)	3592 (51.1)	
**Age group (year)**	**35–45**	1444 (49.5)	2214 (60.5)	3658 (52.0)	0.135
	**46–55**	953 (42.1)	1313 (57.9)	2266 (32.2)	
	**56–65**	447 (40.3)	663 (59.7)	1110 (15.8)	
**Marital status**	**Single**	211 (29.6)	501 (70.4)	712 (10.1)	<0.001
	**Married**	2633 (41.6)	3689 (58.4)	6322 (89.9)	
**Level of education**	**Illiterate**	500 (35.5)	910 (64.5)	1410 (20.0)	<0.001
	**1–5 years**	1015 (37.8)	1668 (62.2)	2683 (38.1)	
	**6–9 years**	581 (45.6)	692 (54.4)	1273 (18.1)	
	**10–12 years**	449 (43.5)	583 (56.5)	1032 (14.7)	
	***>*12 years**	299 (47.0)	337 (53.0)	636 (9.0)	
**BMI**	**< 18.9**	24 (16.7)	120 (83.3)	144 (2.0)	<0.001
	**19–24.9**	693 (31.1)	1538 (68.9)	2231 (31.7)	
	**25–29.9**	1517 (47.0)	1708 (53.0)	3225 (45.8)	
	**30–34.9**	610 (42.5)	824 (57.5)	1434 (20.4)	
**Smoking status**	**No**	2126 (30.3)	3491 (49.7)	5617 (80.0)	<0.001
	**Current**	489 (7.0)	420 (6.0)	909 (12.9)	
	**Former**	224 (3.2)	274 (3.9)	498 (7.1)	

* Test: Chi-Square

**Fig. 1:**
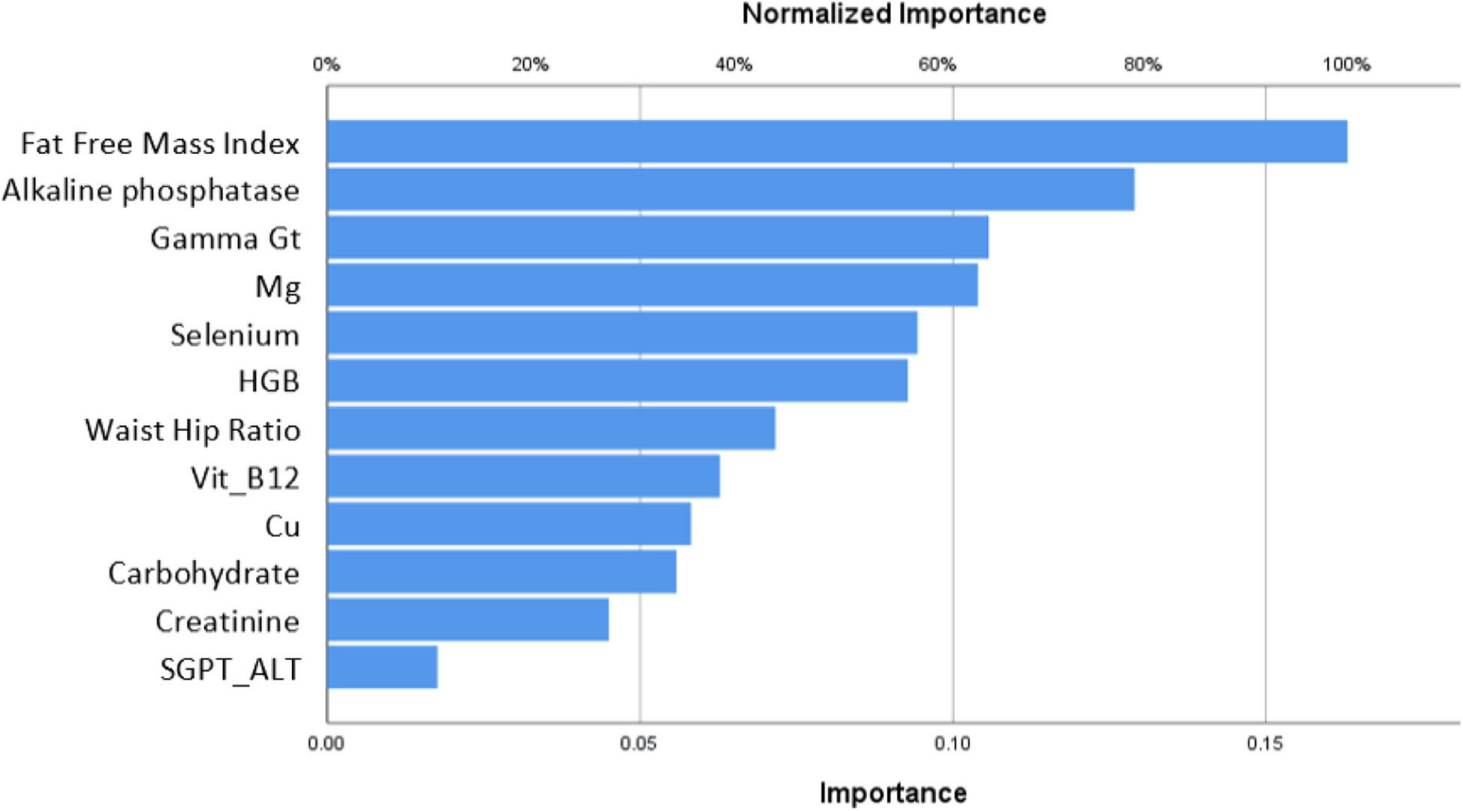
The order of significance of the remaining variables in the ANN model based on the final sensitivity analysis

In the 33-step ANN analysis, the variables with a low significance were removed and at least 12 variables were selected as predictors of the risk of dyslipidemia. As for the variables’ rate of significance in the sensitivity analysis, two of the 12 variables were the most significant, namely the FFM index and alkaline phosphatase, in respective order. The next most important factors involved in the incidence of dyslipidemia were: GGT, magnesium and selenium intake, blood hemoglobin, waist-hip ratio, B12 intake, copper and carbohydrate levels, creatinine levels, and SGPT-ALT (Fig. 1).

Using the 12 aforementioned variables, the ANN efficiency in predicting dyslipidemia was measured based on its accuracy (64.1%), sensitivity (43.1%), specificity (78.9%), and area under the ROC curve (69.0%). After adding the HEI and physical activity level to the other variables, as suggested by the researchers, stepwise multiple regression was used to find the relationship between these 14 variables and the seven lipid markers (i.e. TC, TG, HDL, LDL, TC/HDL, TG/HDL, and LDL/HDL).

According to these models, significant relationships were found for cholesterol and HDL with six variables, LDL with seven variables, triglyceride and LDL/HDL with eight variables, and TC/HDL and TG/HDL with nine variables. The greatest coefficients of determination belonged to the TG/HDL (0.203) and TC/HDL (0.188) model with nine variables and the LDL/HDL (0.180) model with eight variables (Table 2).

**Table 2:** Significant variables in the regression models with the coefficients of the variables, probability, and coefficients of determination

Dependent Variable	independent Variable	β	steps	R^**2**^	***P***
Cholesterol	Waist Hip Ratio	0.171	1	0.028	<0.001
	Alkaline phosphatase	0.091	2	0.046	<0.001
	Gamma Gt	0.109	3	0.056	<0.001
	FFM Index	−0.125	4	0.064	<0.001
	HGB	0.124	5	0.076	<0.001
	Carbohydrate	−0.050	6	0.078	<0.001
Triglyceride	FFM Index	0.188	1	0.078	<0.001
	Waist Hip Ratio	0.164	2	0.116	<0.001
	Gamma Gt	0.107	3	0.142	<0.001
	Alkaline phosphatase	0.099	4	0.152	<0.001
	Physical activity	−0.081	5	0.158	<0.001
	HGB	0.084	6	0.164	<0.001
	Carbohydrate	−0.083	7	0.166	<0.001
	Cu	0.050	8	0.167	0.014
	SGPT ALT	0.031	9	0.167	0.024
HDL	FFM Index	−0.353	1	0.141	<0.001
	Alkaline phosphatase	−0.056	2	0.145	<0.001
	Physical activity	0.067	3	0.150	<0.001
	HGB	−0.055	4	0.152	<0.001
	HEI	−0.033	5	0.154	0.003
	SGPT ALT	−0.027	6	0.154	0.023
LDL	Waist Hip Ratio	0.174	1	0.035	<0.001
	HGB	0.139	2	0.052	<0.001
	Gamma Gt	0.105	3	0.065	<0.001
	FFM Index	−0.065	4	0.071	<0.001
	Alkaline phosphatase	0.066	5	0.075	<0.001
	Carbohydrate	−0.048	6	0.077	<0.001
	HEI	−0.028	7	0.078	0.014
TC/HDL	FFM Index	0.215	1	0.102	<0.001
	Alkaline phosphatase	0.120	2	0.134	<0.001
	Waist Hip Ratio	0.140	3	0.155	<0.001
	HGB	0.141	4	0.173	<0.001
	Gamma Gt	0.062	5	0.181	<0.001
	Physical activity	−0.066	6	0.186	<0.001
	SGPT ALT	0.045	7	0.187	0.001
	Carbohydrate	−0.067	8	0.188	0.001
	Cu	0.039	9	0.188	0.049
TG/HDL	FFM Index	0.279	1	0.130	<0.001
	Waist Hip Ratio	0.132	2	0.156	<0.001
	Gamma Gt	0.081	3	0.176	<0.001
	Alkaline phosphatase	0.099	4	0.187	<0.001
	Physical activity	−0.089	5	0.194	<0.001
	HGB	0.086	6	0.200	<0.001
	SGPT ALT	0.039	7	0.201	0.004
	Carbohydrate	−0.073	8	0.202	<0.001
	Cu	0.050	9	0.203	0.011
LDL/HDL	FFM Index	0.209	1	0.101	<0.001
	Waist Hip Ratio	0.147	2	0.129	<0.001
	HGB	0.152	3	0.155	<0.001
	Alkaline phosphatase	0.095	4	0.168	<0.001
	Gamma Gt	0.069	5	0.175	<0.001
	Physical activity	−0.054	6	0.178	<0.001
	Carbohydrate	−0.035	7	0.179	0.002
	SGPT ALT	0.036	8	0.180	0.009

Despite being significant, the SGPT-ALT variable did not change the coefficients of determination in the TG and HDL models in steps nine and six, respectively. The same was true for Cu in the TC/HDL model in step nine (Table 2).

Among the seven studied markers, the waist-hip ratio was the most effective variable with a greater correlation with LDL and cholesterol. For the other five markers (TG, HDL, LDL, TC/HDL, TG/HDL, and LDL/HDL), the FFM index was the most important variable with the greatest correlation.

Alkaline phosphatase, the FFM index, and HGB were three significant variables in the regression models of all the seven blood lipid markers. In other words, these three variables are important predictors for all the seven blood lipid profiles. Physical activity had a significant effect on five blood lipid markers, but it was not significant in the TC and LDL models. The HEI was significant only in the HDL and LDL models (Table 2).

## Discussion

Identifying the risk factors of cardiovascular diseases is clearly an important task, because planning for the prevention of these diseases is impossible without such data. In this study, the Artificial Neural Network (ANN) was used to identify the variables affecting the lipid profile. George et al. used ANN to predict coronary atherosclerosis [28]. In a cohort study, Liu et al. used a multiple linear regression and logistic regression to investigate the relationship between dyslipidemia and various factors [29]. Wang et al. used the ANN model to identify those at a high risk of dyslipidemia and found the sensitivity, specificity and AUC as 90.41, 76.66 and 86.60%, respectively, for the ANN model, and 57.37, 70.91 and 68.60% for the LR model [30].

Four separate markers (TC, TG, LDL, and HDL) and three ratios (TC/HDL, TG/HDL, and LDL/HDL) were used as the determinants of the blood lipid profile in the present research. Other researchers have also used these markers as the predictors of CHD [31–34].

This study showed that alkaline phosphatase and FFM are common predictors of all lipid markers. A study conducted by Beckman et al. showed that triglycerides have a significant negative correlation with liver alkaline phosphatase isozyme [35]. A study by Schubert et al. also showed that FFM affects HDL and TG levels significantly [36]. The findings of these two studies confirm the results of the present research.

In this study, the correlations of physical activity with TG, HDL, TC/HDL, TG/HDL, and LDL/HDL were significant, as in line with the research by Njølstad, based on which high levels of TG and low levels of HDL correlated significantly with the lack of physical activity [37]. However, Delavar reported contradictory results and concluded that dyslipidemia has no relationship with carbohydrates, fat or physical activity [31].

In the present research, the waist-hip ratio had a significant relationship with TC, TG, LDL, TC/HDL, TG/HDL, and LDL/HDL. Kannel also found that, as a measure of fat stored in the abdominal area, the waist-hip ratio is an independent predictor of cardiovascular diseases [38].

The present research showed a relationship between the intake of selenium and dyslipidemia and found selenium to be the 5th most important predictor of dyslipidemia in the sensitivity analysis; eventually, however, this variable did not enter any of the blood lipid models. This finding was inconsistent with the results reported by Su and Laclaustra, who found that high concentrations of serum selenium were associated with an increase in serum LDL and cholesterol in adults in the US [39] and that long term exposure to selenium may be associated with the risk of development of dyslipidemia in older adults [40]. The reason for this disparity may be the different nutritional culture of the Chinese and Americans and the population in the present study.

A significant relationship was also found between hemoglobin and all the seven blood lipid markers and this variable was the 6th most important predictor of dyslipidemia in the sensitivity analysis. The study by Khan showed a correlation between glycosylated HGB, TC, TG, and LDL, on the one hand, and decreased HDL, on the other, which indicates the linear relationship between HGB and dyslipidemia [41]. The study by Ladeia was also indicative of the significant relationship of HGB with dyslipidemia and blood lipid markers [42]. This finding was consistent with the results of the present research.

In this study, vitamin B12 intake was the 8th major predictor of dyslipidemia in the sensitivity analysis, but it had no significant relationship with any of the blood lipid markers; meanwhile, in the study by Mahalle [43], vitamin B12 was an effective predictor of CHD.

In the present study, copper (Cu) was the 9th major predictor of dyslipidemia in the sensitivity analysis and had a significant correlation with TG, TC/HDL and TG/HDL. In line with this study, Tosco [44] also showed the relationship of Cu with dyslipidemia.

Magnesium (Mg) was the 4th important predictor of dyslipidemia in the sensitivity analysis, but it showed no significant correlation with any of the blood lipid markers. Meanwhile, Nasri reported a positive correlation between serum levels of magnesium and TG [45]. This disparity could be due to the differences in the study subjects, who were hemodialysis patients in their study.

As SGPT-ALT did not increase the coefficients of determination in the last step in the TG and HDL models, expensive SGPT-ALT experiments can be removed from TG and HDL models. Since the same is true for the Cu TC/HDL model, it can be excluded as well.

## Strengths and limitations

One of the strengths of this study was the large sample size and the use of many different variables, and one of its weaknesses was the large amount of missing data.

## Conclusion

According to the sensitivity analysis, the most important variables predicting dyslipidemia included the FFM index, alkaline phosphatase, and GGT, with the acceptable accuracy of 64.1%. The best models in terms of the coefficients of determination were the TG/HDL and TC/HDL models with nine variables. Alkaline phosphatase, the FFM index, and HGB were three common predictors for all the lipid markers. Health authorities are therefore recommended to adopt plans to help keep these indices in the normal range in the community so as to help improve lipid levels and prevent related diseases.

## Data Availability

The datasets used and/or analysed during the current study are available from the corresponding author on reasonable request.

## Abbreviations

ANN: Artificial Neural Network
FFM: Fat Free Mass
LDL: Low Density Lipid
HDL: High Density Lipid
CAD: Coronary Artery Disease
CHD: Chronic Heart Disease
BMI: Body Mass Index
GCS: Glasgow Coma Scale
TC: Total Cholesterol
TG: Triglyceride
Mg: Magnesium
Cu: Copper
NHIS: Health Insurance Scheme
SES: Socio-Economic Status
PCA: Principal Component Analysis
HEI: Healthy Eating Index
HGB: Hemoglobin
FFQ: Food Frequency Questionnaires
